# Supplementary material for: Splice-Junction-Based Mapping of Alternative Isoforms in the Human Proteome
Source: Cell Rep. Author manuscript; Available in PMC 2020 Jan 15. (PMC6961840; doi:10.1016/j.celrep.2019.11.026)
Supplement: 2 [file NIHMS1546469-supplement-2.zip › DF1/PXD009737/TestisGluC/TestisGluC_24_RANBP1_DAQKFKTKFEE.pdf]

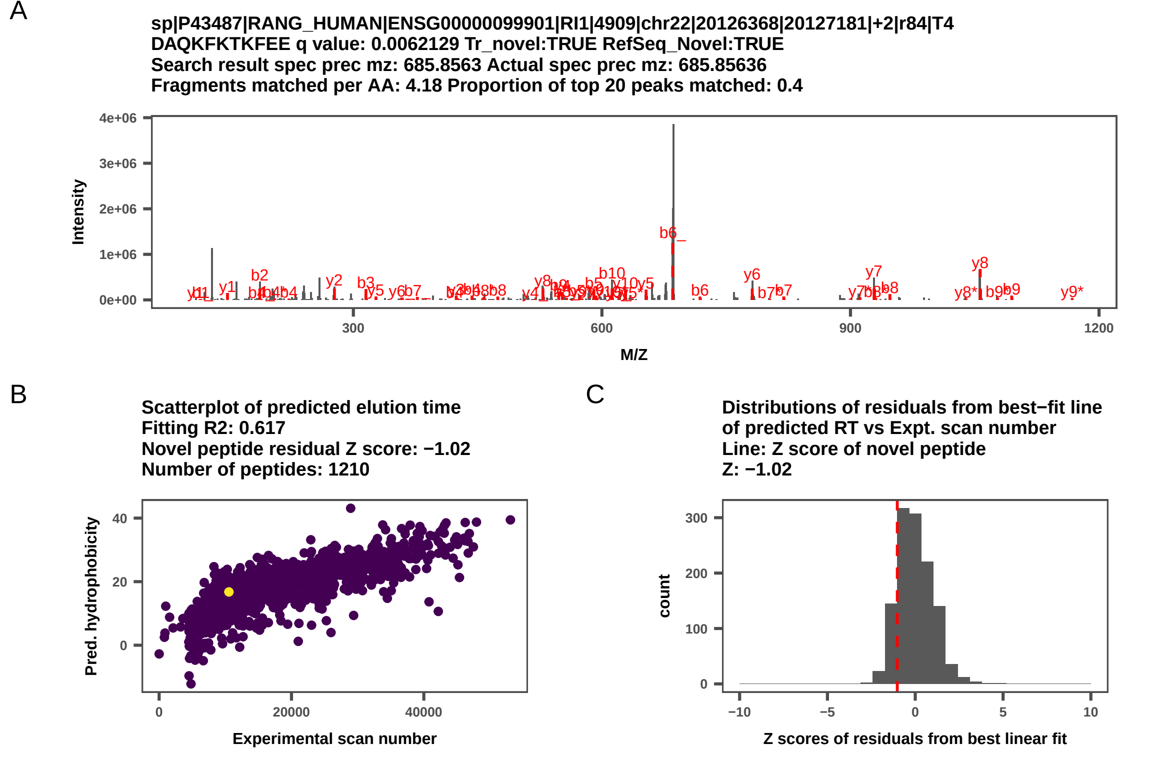
A
sp|P43487|RANG_HUMAN|ENSG00000099901|RI1|4909|chr22|20126368|20127181|+2|r84|T4
DAQKFKTKFEE q value: 0.0062129 Tr_novel:TRUE RefSeq_Novel:TRUE
Search result spec prec mz: 685.8563 Actual spec prec mz: 685.85636
Fragments matched per AA: 4.18 Proportion of top 20 peaks matched: 0.4
Intensity
4e+06
3e+06
2e+06
1e+06
0e+00
M/Z
300
600
900
1200
b6_
y8
y7
y6
b10
y8
y2
b3
b2
y1
b6
y9*
B
Scatterplot of predicted elution time
Fitting R2: 0.617
Novel peptide residual Z score: −1.02
Number of peptides: 1210
Pred. hydrophobicity
40
20
0
0
20000
40000
Experimental scan number
C
Distributions of residuals from best−fit line
of predicted RT vs Expt. scan number
Line: Z score of novel peptide
Z: −1.02
count
300
200
100
0
−10
−5
0
5
10
Z scores of residuals from best linear fit
